# Combined Carbon Dioxide Laser with Photodynamic Therapy for Nodular Basal Cell Carcinoma Monitored by Reflectance Confocal Microscopy

**DOI:** 10.3390/medicina60010030

**Published:** 2023-12-24

**Authors:** Antonio Alma, Linda Pongetti, Alessandro Clementi, Johanna Chester, Matteo Toccaceli, Silvana Ciardo, Elena Zappia, Marco Manfredini, Giovanni Pellacani, Maurizio Greco, Luigi Bennardo, Francesca Farnetani

**Affiliations:** 1 Dermatology Unit, Surgical, Medical and Dental Department of Morphological Sciences Related to Transplant, Oncology and Regenerative Medicine, University of Modena and Reggio Emilia, 41125 Modena, Italy; antonioalma@virgilio.it (A.A.); pongettilinda@gmail.com (L.P.); dr.alessandroclementi@gmail.com (A.C.); johanna.chester@gmail.com (J.C.); toccaceli94@gmail.com (M.T.); silvana.ciardo@unimore.it (S.C.); marco.manfredini@unimore.it (M.M.); mauriziogreco77@hotmail.com (M.G.); farnetani.francesca@gmail.com (F.F.); 2 Department of Health Sciences, Magna Graecia University, 88100 Catanzaro, Italy; elena.zappia@hotmail.it; 3 Dermatology Clinic, Department of Clinical Internal, Anesthesiological and Cardiovascular Sciences, Sapienza University of Rome, 00185 Rome, Italy; pellacani.giovanni@gmail.com

**Keywords:** CO_2_ laser, photodynamic therapy, basal cell carcinoma, reflectance confocal microscopy

## Abstract

*Introduction*: Basal cell carcinoma (BCC) represents around 80% of all malignant skin cancers worldwide, constituting a substantial burden on healthcare systems. Due to excellent clearance rates (around 95%), surgery is the current gold-standard treatment. However, surgery is not always possible or preferred by patients. Numerous non-surgical therapies, sometimes combined, have been associated with promising tumor free survival rates (80–90%) in non-melanoma skin cancers (NMSCs). Most research has enrolled superficial basal cell carcinomas (sBCCs), with limited recent studies also involving low-risk nodular BCCs (nBCCs). Given lower efficacy rates compared to surgery, close monitoring during the follow-up period is essential for patients treated with non-surgical therapies. Monitoring with dermoscopy is constrained by low sensitivity rates. Reflectance confocal microscopy (RCM) is more sensitive in monitoring non-surgically treated NMSCs. *Case presentation*: A 41-year-old woman with a single nBCC relapse following photodynamic therapy (PDT) located on the dorsum of the nose presented to our center. Given the aesthetically sensitive location of the lesion and the patient’s preference for a non-surgical approach, a combined treatment of CO_2_ laser and PDT was prescribed. A superpulsed CO_2_ laser (power: 0.5–3 W, frequency: 10 Hz, spot size 2 mm) with two PDT sessions (2 weeks apart) were conducted. At 6 weeks follow-up, monitoring performed with RCM revealed a reduction but not eradication of basaloid tumor islands. Another 2 sessions of PDT were recommended. At 3, 12 and 30 months of follow-up, the nasal dorsum area of the previous nBBC lesion was noted to be slightly hypopigmented (observed clinically), with a mild erythematous background (observed by dermoscopy). RCM evaluation confirmed the absence of RCM BCC criteria. The cosmetic outcome was very much improved. *Conclusions*: Combined CO_2_ laser and PDT for the treatment of a localized nBCC on the dorsum of the nose of a 41-year-old proved to offer tumor free survival at 30-month follow-up, as monitored with RCM. RCM is useful for the evaluation of non-surgical therapies as it has comparably higher sensitivity than dermoscopy and is especially useful in cases of suspected late recurrence. Further studies are needed to validate ongoing tumor free survival following this combined nonsurgical approach in the treatment of nBCC.

## 1. Introduction

Basal cell carcinoma (BCC) stands as the most prevalent malignancy worldwide, representing around 80% of all malignant skin cancers [1]. BCCs typically manifest as slowly developing nodules with a shiny surface, with occasional ulceration in the center of the lesion [2]. Undiagnosed BCCs can progress, with locally destructive tumor growth and invasion of surrounding tissues. However, metastases associated with BCC are rare and mortality is contained [2]. There is a significant age-related incidence of BCC, with the most important recent incremental trend observed among young European women [3]. BCC constitutes a substantial burden on healthcare systems, demanding attention and strategic approaches for a continuous exploration of innovative therapeutic modalities. 

Appropriate treatment is essential to minimize morbidity associated with local skin and tissue damage [4]. Currently, surgical therapy is the gold standard treatment, with clearance rates of more than 95% [5]. Excisional techniques, including Mohs micrographic surgery, continue to ensure high rates of tumor eradication, with minimal damage to surrounding healthy tissue. However, some patients are unsuitable candidates for surgery due to contraindications, such as advanced age and severe comorbidities, or may refuse a surgical approach, especially in the case of lesions located in cosmetically sensitive areas. Tumor characteristics, such as multiple lesions, large size, or tumor location, can also complicate a surgical approach [6]. Further, risks of surgical excision, including scarring, tissue defect, longer convalescence and increased risk of infection, are to be taken into consideration [7]. Therefore, despite the efficacy of surgical options, the rising incidence of BCCs endorses the necessity for research into alternative interventions.

Non-surgical modalities have emerged as attractive alternatives for BCC treatment due to inferior invasiveness. Approaches include radiation therapy, cryotherapy, imiquimod, carbon dioxide (CO_2_) laser and photodynamic therapy (PDT). Studies assessing the efficacy of non-surgical modalities report clearance rates for non-melanoma skin cancer (NMSC) of around 80–90%. However, most BCC lesions included in these studies are of the superficial subtype (sBCC) [8]. Further, the absence of intraoperative histopathological examination to confirm complete removal of the neoplasm and ongoing tumor clearance casts doubt on the efficacy of non-surgical approaches [9].

PDT requires the application of a topical photosensitizing agent which is then activated by light. PDT selectively targets cancerous cells [10] and has applications beyond primary treatment, demonstrating efficacy in managing recurrent BCCs. PDT’s efficacy is related to the site, thickness and histological subtype of the lesion. The most effective response is for sBCC, with total remission rates of around 90%. However, due to PDT’s limited depth of penetration (2 mm absorption) its effectiveness of treatment of nodular BCCs (nBCCs) is limited [2]. Small nBCC show relatively good response probabilities of around 80% [11]. However, PDT therapy can have adverse effects, including pain during or after treatment, erythema and failure of long-term wound healing with exudation [12].

Another non-surgical avenue for BCC management harnesses the precision of laser technology. The carbon dioxide (CO_2_) laser produces an emission in the far-infrared spectrum (10,600 nm)based on a mixture of gases; carbon dioxide, helium and nitrogen. Due to its high affinity for intra- and extra-cellular water, the CO_2_ laser enables precise tissue ablation with minimal thermal diffusion [13]. CO_2_ lasers can be used with different pulses, enabling the combination of ablative and thermal properties to optimize vaporization and biostimulation [9]. Several case series have demonstrated the efficacy of the superpulsed CO_2_ laser in the treatment of BCC, mainly in candidates ineligible for a surgical approach or with multiple lesions [14]. Direct adverse effects of laser therapy include pain, redness and oedema, especially associated with older methods using stronger parameters. With the new “superpulsed” and scanned CO_2_ systems, adverse effects have been reduced; most are resolved within hours of treatment. In rare cases, hypo- and hyper-chromia have been reported, particularly when treatments are performed during the summer months [9]. 

The combination of CO_2_ laser and PDT seem promising, but limited data are available [6,15]. A study including 13 nBCCs (nBCC) in 12 patients, reported a 100% clearance rate at 3 month follow-up [15]. At longer follow-up (mean 10.7 months) clearance rates were reported to be 97.2%, for both nBCC and sBCC [6]. This rate was confirmed at 32 months of follow up in a study of 177 BCCs with histopathologically proven superficial, nodular, infiltrative, morpheic and mixed BCCs [16] (see Table 1).

Most monitoring is performed with dermoscopy, due to the simplicity, speed and cost-effectiveness of the evaluation. Dermoscopic criteria for BCC have been extensively reported in literature [2]. Features include arborising vessels, superficial telangiectasias, multiple erosions, ulceration, blue-grey clods of different dimensions, leaf-like areas, spoke-like areas, concentric structures, structureless white areas, white clods and multiple aggregated yellow-white globules. However, effectiveness of dermoscopy for BCC identification and monitoring are questionable, as the criteria for BCC identification are shared with other lesion type, such as atypical vessels which is a shared pattern with melanoma [17]. As a result, dermoscopy can return false-negative results [18]. For example, structureless white areas and superficial fine telangiectasias may be erroneous positive features, as their presence does not necessarily indicate tumor persistence [19]. Additionally, dermoscopic images vary according to skin tone [20].

As the emphasis on the importance of early and accurate diagnosis grows, efforts for improved patient monitoring have expanded. Reflectance confocal microscopy (RCM) is a non-invasive imaging technique that enables real-time visualization of cellular structures. By means of high sensitivity and specificity, RCM significantly impacts the patient clinical course through enhanced diagnostic precision [21]. RCM acquires en-face images of the epidermis and papillary dermis, with a resolution that can be compared to that of histology [22]. Due to the distinct refractive indexes of skin tissue, black-and-white images are generated [23]. RCM criteria for BCCs have been established in the literature, including the presence of basaloid tumor islands (which appear as tightly packed cells surrounded by fissures and may be organized as cords), hypo-refractile zones with a “festooned contour” outlined by highly reflective collagen (the so-called “dark silhouettes” that represent basaloid islands lacking pigment), presence of elongated polarized basaloid nuclei present in the epidermis, inflammatory cells and increased dermal vasculature [24]. Studies have reported RCM sensitivity and specificity rates of 100% and 72.5% for NMSC monitoring of non-surgically treated lesions [1,18] and a reduction of unnecessary biopsy in BCC lesions considered equivocal with dermoscopy [25].

We report a case of a nBCC located on the nasal dorsum, successfully treated with combined CO_2_ laser and PDT, monitored with RCM evaluation over a 30-month period.

## 2. Case Report

**Figure 1:**
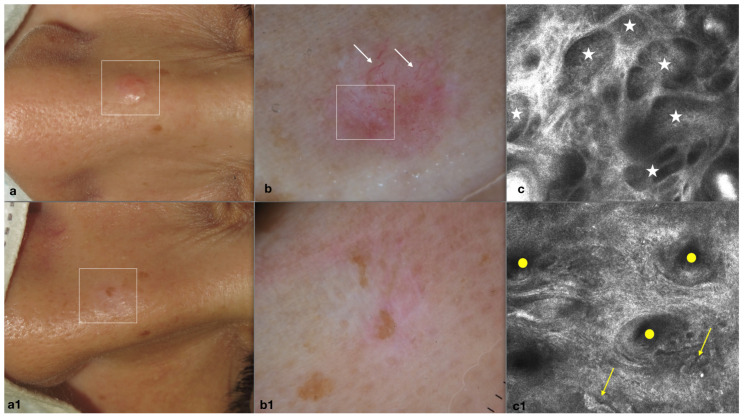
(**a**) Clinical image of a nodular basal cell carcinoma (nBCC) located on the nasal dorsum of 41-year-old woman at baseline. (**b**) Pre-operative dermoscopic image of nBCC showing short fine teleangiectasia (white arrows) and shiny white-red structureless areas (white square area). (**c**) Reflectance confocal microscopy (RCM) image shows basaloid tumor islands (white stars) of the nBCC at baseline. (**a1**) Complete clinical healing of the lesion at 30 months follow-up. (**b1**) Dermoscopic clearance and (**c1**) evidence of polycyclic papillary contours (yellow arrows) and follicular openings (yellow circles) confirming RCM clearance.

A 41-year-old female with a single, relapsed nBCC on the nasal dorsum presented to our center requiring medical attention. A shave biopsy performed at a local outpatient clinic two years prior, had established a BCC diagnosis. Treatment of two PDT sessions had been undertaken by our colleagues. Clinical examination at our center revealed a recurrent nodular lesion (5 mm in diameter). Dermoscopy feature observations included typical arborizing vessels and shiny white–red structureless areas. RCM confirmed nBCC detection through the observation of basaloid tumor islands (Figure 1). 

The patient was informed about treatment options and expressed a preference for a non-surgical treatment approach. This was mainly due to the aesthetically sensitive location of the lesion. Given the ineffectiveness of prior PDT alone, a combined treatment of CO_2_ laser and PDT was arranged. 

Local anesthesia with mepivacaine 2% was injected around the lesion. A super-pulsed CO_2_ laser (power: 0.5–3 W, frequency: 10 Hz, spot size 2 mm) was employed. After the vaporization of the nodule, the carbonized residue was removed with saline-soaked gauze. The procedure was repeated until it reached the reticular dermis. A topical antibacterial ointment was prescribed twice daily for ten days. 

At two weeks of follow-up, wound re-epithelialization and the flattening of the lesion were clinically confirmed. Two PDT sessions were performed (at a distance of 2 weeks apart), two weeks after the CO_2_ laser intervention. The photosensitizing agent methyl aminolevulinate (MAL) application, covered with an occlusive dressing, prevented light penetration for 3 h. The area was irradiated with a red light-emitting diode (LED) lamp (Aktilite CL128^®^, Galderma SA, Lausanne, Switzerland) using a total dose of 37 J/cm^2^, delivered over 8 min. Treatment was well tolerated, with mild pain and localized erythema reported. The application of an antibiotic topical ointment was prescribed for seven days. 

At six weeks of follow-up, monitoring performed with RCM (Vivascope 1500, Caliber ID, Rochester, NY, USA) revealed a reduction in the number of basaloid tumor islands. Clinicians and the patient decided to undertake two additional PDT sessions. 

At the 3, 12 and 30-month follow-ups, the nasal dorsum area of the previous nBBC lesion was noted to be slightly hypopigmented (observed clinically) and with a slight erythematous background (observed with dermoscopy). RCM evaluation confirmed the absence of BCC RCM criteria. The cosmetic result, assessed by clinicians and the patient with a five-grade global aesthetic improvement scale, was classified as “very much improved”, see Figure 1.

## 3. Discussion and Conclusions

The combined CO_2_ laser and PDT approach for treating a localized nBCC on the dorsum of the nose of a 41-year-old female patient, monitored with RCM, revealed ongoing tumor-free survival at 30 months follow-up. BCC was identified at baseline through the observation of standard RCM BCC criteria of basaloid tumor islands [26]. The patient expressed the preference for a minimally invasive procedure for oncological radicality, mainly due to the cosmetically sensitive location of the lesion. RCM for therapeutic efficacy assessment was used given the superior performance compared to other less sensitive or more invasive imaging techniques. 

As compared to flat lesions, the efficacy of dermoscopy to differentially diagnose nBCC from nodular melanoma and dermal naevi is limited [17]. Unnecessary biopsy, misdiagnosis or unnecessary surgical excision frequently occur. RCM has been acknowledged as a potential tool for early BCC diagnosis [25,27]. A recent randomized, multicenter trial including multiple BCC subtypes reported similar overall BCC diagnostic sensitivity of RCM compared to punch biopsy, but a significantly lower sensitivity [28]. However, given the known limited depth penetration of the device (200 µm), restricting visualization by hyperkeratosis and ulceration, the applicability of RCM to nBCC diagnosis is questionable. In our case report, RCM assessment of the BCC lesion enabled visualization of clear BCC criteria (basaloid tumor islands), facilitating lesion recognition [27]. Given the aesthetically sensitive location of the lesion on the face of a young woman, alternative therapies to surgery were considered.

Most studies investigating alternative therapies to surgery have included sBCCs only. Current non-surgical approaches comprise radiation therapy, cryotherapy, imiquimod, carbon dioxide (CO_2_) laser and PDT [6]. Therefore, research into alternative treatments to surgery for nBCC, especially for patients who are ineligible for surgery, refuse surgery or have lesions located in aesthetically complex locations, are restricted. Treatment with 5% imiquimod in low risk nBCC have proven less effective than surgical approaches [29,30]. Imiquimod is a topical, non-invasive treatment for NMSC, which targets toll-like receptor 7 (TLR-7) on antigen-presenting cells, triggering both innate and adaptive immune responses. Clinically, the treated area is characterized by an initial strong inflammatory response but the final cosmetic outcome is usually very good [31]. However, cutaneous side effects from the topical application of imiquimod include pityriasis rubra pilaris, psoriasiform eruptions, pemphigus-like lesions, erythema multiforme, subacute lupus, lichen planus and vitiligo-like depigmentation [32]. Radiation therapy has also been experimented in low-risk nBCCs, with data suggesting this approach to be a valid alternative to surgery. The main limitation of radiation therapy is the protracted patient commitment to multiple patient visits over several weeks [30,33]. Cryotherapy, tissue destruction through freezing, is widely adopted in outpatient services due to the combination of safety, efficacy, low cost and ease of use [34]. Common local reactions to cryotherapy include vesicles, erythema, oedema and hypopigmentation and hypertrophic scarring. Severe and painful hemorrhagic vesicles and/or bullae occur rarely, however, these side effects can open the way to secondary infections [35]. These techniques are sometimes applied as adjuvant therapy for incompletely excised BCCs. However, efficacy data are incomplete [36].

Combination of these therapies has been experimented. Kim et al. [37] treated 8 BCCs with CO_2_ laser following PDT. El-Khalawany et al. [38] assessed the efficacy of combining ablative CO_2_ laser, imiquimod 5% and diclofenac 3% in 14 high-risk and inoperable BCC. Sutedja et al. used a combination of cryotherapy and imiquimod 5% to treat sBCC located on the torso of the patient [39].

The combination of CO_2_ laser and PDT therapy for BCC non-surgical treatment is, therefore, not novel. Several studies have reported positive CO_2_ laser/PDT combined therapy outcomes for both sBCC and limited nBCC. However, post-treatment monitoring has been exclusively reported according to dermoscopy and clinical appearance [6,15,16]. While the employment of clinical and dermoscopy monitoring techniques are simple, fast and inexpensive, estimates suggest that as many as 20% false negatives can be observed at dermoscopy [18,19,40,41]. Our case report is intended to enhance current knowledge of combined CO_2_ laser and PDT therapy for, the less frequently reported, nBCC. Larger and more detailed experiences with alternative approaches in nBCC need to be shared in literature to assess efficacy and safety for this patient cohort.

As patients treated with non-surgical modalities do not have histological proof of disease recovery, long term post-treatment surveillance is necessary [24]. The greatest diagnostic sensitivity and specificity of the tool is of fundamental importance for adequate and safe follow-up. The integration of RCM into monitoring practices increases diagnostic sensitivity and is therefore emerging as a valuable adjunctive follow-up tool. Combining the strengths of RCM with established methods, such as dermoscopy, can offer a more comprehensive understanding of skin lesions and enhanced diagnostic accuracy.

Despite higher costs and training requirements for image interpretation, RCM’s improved sensitivity and specificity in BCC detection following non-surgical treatment compared to dermoscopy and clinical evaluations [18] and almost comparable performance with histopathology [1,18], make non-invasive and routine clearance monitoring possible. RCM enables low invasive and timely assessments, avoiding techniques of biopsy and subsequent histological confirmation. However, given the barriers to the adoption of RCM, including initiation costs of the device, operator training and required experience, the generalizability of RCM monitoring of patients is currently restricted [42]. 

Our case study is inherently limited by the outcome observed in a single individual, without histopathological confirmation of tumor eradication or any investigations into the molecular biology behind the treatment. Our study is limited to a description of the therapeutic process and follow up only. However, long term follow-up of tumor clearance (30 months) observed with RCM reported in this case and others in the literature, warrant further studies into the validation of long-term efficacy of this combined approach for nBCC. Such investigations will contribute to the integration of this combined non-surgical approach into routine dermatological practice.

The efficacy of combined CO_2_ laser and PDT for nBCC and the ongoing tumor free survival at 30 months in our case study, was confirmed with RCM. RCM proved useful in both baseline BCC identification and post-operative monitoring. Continued research into the efficacy of alternative, non-surgical therapies for nBCC are essential for comparative assessments of applicability of these techniques in routine dermatological practice.

## Figures and Tables

**Table 1:** Data collection synthesis of the main articles discussing cases of basal cell carcinoma treated with a combined approach using photodynamic therapy and CO_2_ laser.

Authors	Number of BCCs	BCC Histological Type	BCC Location	Techique for Monitoring	Follow-Up Period, Months (Range)
Ferrara et al. [6].	181	46 sBCCs 135 nBCCs	sBCCs: 24 trunk 15 limbs 7 head and neck nBCCs: 84 head and neck 35 trunk 16 limbs	Clinical and dermoscopic. Doubtful case were subjected to biopsy	10.7 (4–18)
Whitaker et al. [15]	13	13 nBCC	Head and neck	Clinical	18.1 (7–26)
Shokrollahi et al. [16]	177	74 BCC (subtype unspecified) 34 sBCC, 50 Nbcc, 9 infiltrative 7 morphenic 3 mixed	Head and neck (mainly)	Clinical	32.2 (8–69)

sBBC, superficial basal cell carcinoma; nBCC, nodular basal cell carcinoma [6,15,16].

## Data Availability

Data available from the corresponding author upon reasonable request.
